# Identification and structural insight of an effective PPARγ modulator with improved therapeutic index for anti-diabetic drug discovery†
†Electronic supplementary information (ESI) available: ESI includes experimental parts, copies of ^1^H and ^13^C NMR spectra of products, Fig. S1, Tables S1–S4 and validation report PDF file. See DOI: 10.1039/c9sc05487a


**DOI:** 10.1039/c9sc05487a

**Published:** 2020-01-21

**Authors:** Haowen Jiang, X. Edward Zhou, Jingjing Shi, Zhi Zhou, Guanguan Zhao, Xinwen Zhang, Yili Sun, Kelly Suino-Powell, Lei Ma, Hui Gao, Xiyong Yu, Jia Li, Jingya Li, Karsten Melcher, H. Eric Xu, Wei Yi

**Affiliations:** a Guangzhou Municipal and Guangdong Provincial Key Laboratory of Protein Modification and Degradation & Molecular Target and Clinical Pharmacology , State Key Laboratory of Respiratory Disease , School of Pharmaceutical Sciences & the Fifth Affiliated Hospital , Guangzhou Medical University , Guangzhou , Guangdong 511436 , China . Email: yiwei@gzhmu.edu.cn; b VARI/SIMM Center , Center for Structure and Function of Drug Targets , CAS-Key Laboratory of Receptor Research , Shanghai Institute of Materia Medica , Chinese Academy of Sciences , Shanghai 201203 , China . Email: eric.xu@simm.ac.cn; c National Center for Drug Screening , State Key Laboratory of Drug Research , Shanghai Institute of Materia Medica , Chinese Academy of Sciences , Shanghai 201203 , China . Email: jyli@simm.ac.cn ; Email: jli@simm.ac.cn; d Structural Biology Program , Center for Cancer and Cell Biology , Van Andel Research Institute , Grand Rapids , Michigan 49503 , USA

## Abstract

Peroxisome proliferator-activated receptor γ (PPARγ) is a key regulator of glucose homeostasis and lipid metabolism, and an important target for the development of modern anti-diabetic drugs. However, current PPARγ-targeting anti-diabetic drugs such as classical thiazolidinediones (TZDs) are associated with undesirable side effects. To address this concern, we here describe the structure-based design, synthesis, identification and detailed *in vitro* and *in vivo* characterization of a novel, decanoic acid (DA)-based and selective PPARγ modulator (SPPARγM), VSP-77, especially (S)-VSP-77, as the potential “hit” for the development of improved and safer anti-diabetic therapeutics. We have also determined the co-crystal structure of the PPARγ ligand-binding domain (LBD) in complex with two molecules of (S)-VSP-77, which reveal a previously undisclosed allosteric binding mode. Overall, these findings not only demonstrate the therapeutic advantage of (S)-VSP-77 over current TZD drugs and representative partial agonist INT131, but also provide a rational basis for the development of future SPPARγMs as safe and highly efficacious anti-diabetic drugs.

## Abstract 2

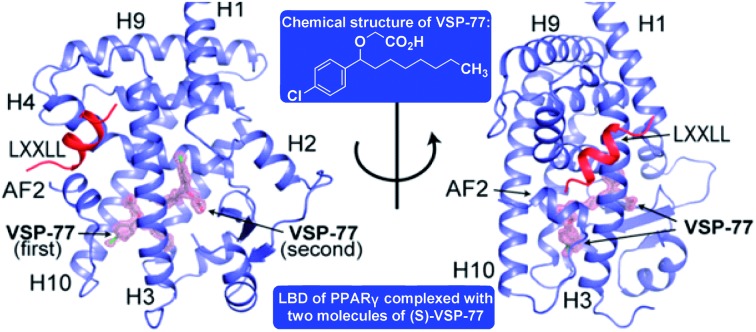
A novel and potent “hit” VSP-77, especially (S)-VSP-77, has been identified as the effective PPARγ modulator for anti-diabetic drug discovery.

## Introduction

Type 2 diabetes mellitus (T2DM), also known as non-insulin-dependent diabetes mellitus, accounts for >90% of all cases of diabetes. This condition is characterized by high blood glucose (hyperglycemia) mainly resulting from resistance to insulin in peripheral tissue.^1^ One of the most remarkable pathological features in diabetic patients is energy surplus-generated obesity. Adipose tissue is the largest lipid and energy storage in human body. However, during obesity, adipose tissue might become severely dysfunction and fail to appropriately expand to store the surplus energy. These conditions lead to ectopic fat accumulation in other tissue, and progressive insulin resistance and T2DM.^2^–^4^ Therefore, it is crucial to target to the improvement of adipose dysfunction for regulating energy homeostasis and obesity.

PPARγ is a master regulator of adipose cell differentiation and development that belongs to the nuclear hormone receptor superfamily.^5^–^8^ PPARγ is also the target receptor for the TZD class of anti-diabetic drugs, which act as PPARγ full agonists *via* an activation function 2 (AF-2)-mediated “lock” mechanism. TZDs such as rosiglitazone (Rosi) have been widely used for the treatment of T2DM by lowering glucose levels and improving insulin sensitivity.^9^,^10^ However, despite their excellent potencies in treating diabetes, they possess many severe side effects such as fluid retention, weight gain, cardiac hypertrophy, and hepatotoxicity in the clinic.^11^–^16^ Due to these side effects, Rosi has been withdrawn from the European market. Recently, pioglitazone, the most widely used TZD, has also been associated with controversial side effects including bladder cancer.^17^ Undoubtedly, there is an urgent need to discover new, safe and highly efficacious PPARγ ligands with improved therapeutic profiles.

An alternative approach has been taken to seek for non-TZD PPARγ partial agonists, also known as SPPARγMs. SPARγMs stabilize the AF-2 helix in distinct states between closed and open conformations, which allows AF-2 to more selectively recruit co-activators, which is associated with reduced side effects relative to TZD compounds.^18^–^24^ As a consequence, a large number of both naturally occurring and synthetic non-TZD PPARγ partial agonists/SPPARγMs have been reported.^25^–^33^ Among them, carboxylic acid derivatives have attracted considerable attention.^34^–^46^ For example, Miyachi and co-workers reported a class of optically active α-benzylphenylpropanoic acids as potent SPPARγMs.^34^ Previously, our group also disclosed naturally occurring DA as a direct ligand of PPARγ with better pharmacological properties, such as the diminished ability to induce adipocyte differentiation.^46^ The crystal structure of PPARγ bound with DA (PDB code ; 3U9Q) revealed that DA occupied a novel binding site and only partially activated PPARγ by only weakly stabilizing the AF-2 helix.^46^,^47^ Further structural analysis identified a region of the hydrophobic pocket near the γ-position of DA that could be exploited for future design (Fig. S1†). However, due to its low affinity and poor selectivity for PPARγ, DA's efficacy in decreasing glucose levels in mice was less significant than that of Rosi. Moreover, pharmacokinetic studies showed that the β-position of DA could be readily oxidized and subsequently broken *in vivo*,^48^,^49^ which might lead to further loss of glucose lowering efficiency.

By using the DA-bound-PPARγ LBD structure as a template, we designed new DA-based molecules by introducing an oxygen atom in the β-position and variable moieties in the γ-position of DA, which we hypothesized would occupy the newly identified sub-pocket, and thereby increase the affinity and selectivity toward PPARγ. Increased complex stability in turn might lead to improved glucose-lowering capability and reduced side effects. In order to confirm this hypothesis, several DA-based compounds were constructed. To our delight, a novel compound, VSP-77, was identified as a potent SPPARγM with desired pharmacological properties. Therefore, in this paper, we describe the detailed biological characterization of VSP-77 and its active form (S)-VSP-77 *in vitro* and *in vivo* for anti-diabetic drug evaluation, and then revealed the unique binding mode of (S)-VSP-77 to PPARγ LBD through co-crystal structural analysis. Together, our results demonstrate that (S)-VSP-77 can serve as a promising candidate for T2DM therapy and establish a rational foundation for designing specific drugs targeting PPARγ with advantages over current TZD drugs and representative partial agonist INT131.

## Results

### The synthesis of VSP-77

**Fig. 1:**
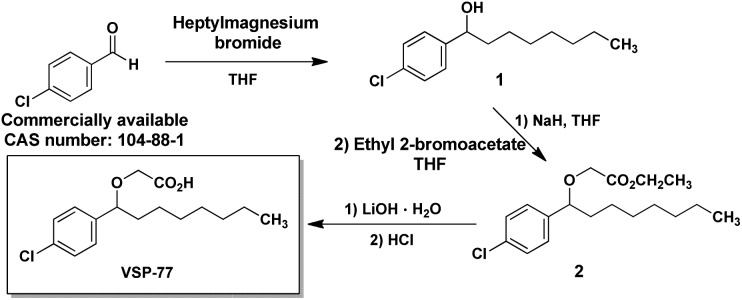
The synthetic routes of VSP-77.

VSP-77 was synthesized in three steps as shown in Fig. 1: a classical nucleophilic addition (Grignard reaction) between heptylmagnesium bromide and 4-chlorobenzaldehyde, subsequent etherification and final hydrolysis. The Grignard reaction proceeded with 85% yield to form the alcohol **1**. Etherification of the intermediate alcohol **1** with ethyl 2-bromoacetate gave the ethyl ester **2** in a moderate yield (45%). Hydrolysis of ethyl ester **2** in the presence of lithium hydroxide hydrate provided the desired VSP-77 with a yield of 80% (see the ESI† for ^1^H NMR and ^13^C NMR spectra).

### The synthesis of (R)-VSP-77 and (S)-VSP-77

**Fig. 2:**
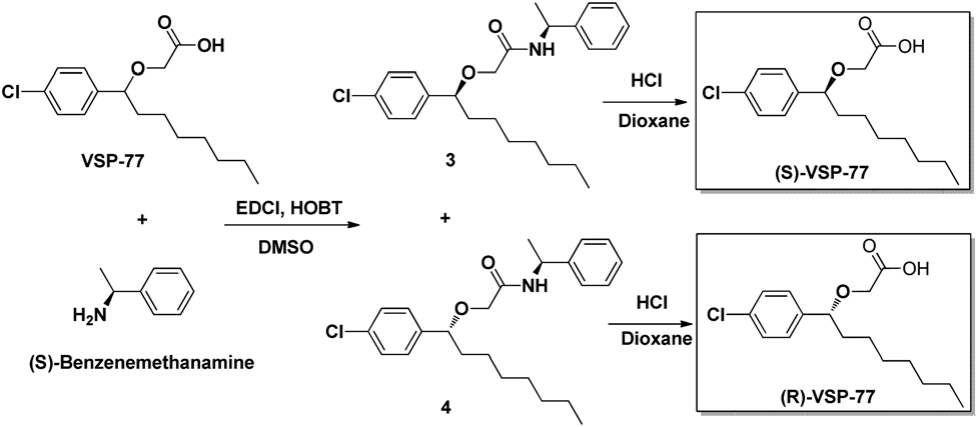
The synthetic routes of (R)-VSP-77 and (S)-VSP-77.

(R)-VSP-77 and (S)-VSP-77 were respectively synthesized in two steps as demonstrated in Fig. 2: Firstly, a facile condensation in the presence of EDCI and DMSO to provide amides 3 and 4 in 42% and 37% yields, respectively, followed by ether hydrolysis assisted by 6 N HCl solution to afford the corresponding products (R)-VSP-77 and (S)-VSP-77 in decent yields.

### Identification of VSP-77 as a potent PPARγ ligand

**Fig. 3:**
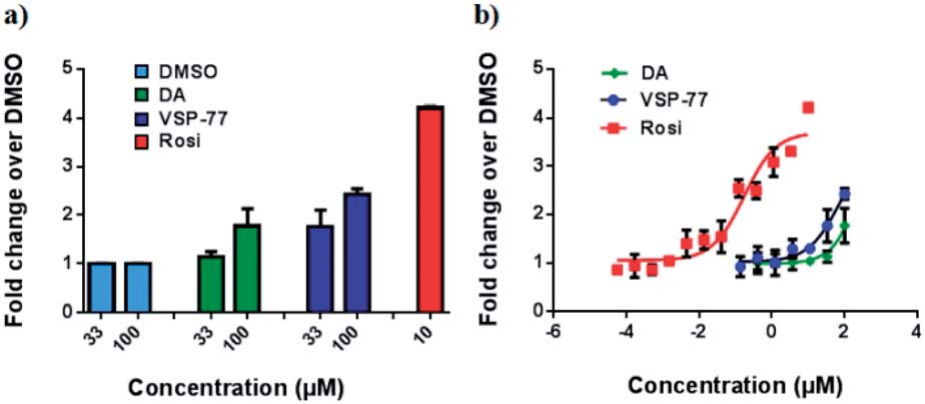
Luciferase reporter assay of PPARγ activation in Cos-7 cells. The luciferase activity was normalized against renilla luciferase units. Fold activation was calculated against DMSO with no ligand treatment. (a) Fold activation in response to 33 μM and 100 μM of DA and VSP-77, and 10 μM of Rosi; (b) dose–response curve. The concentration of Rosi used ranged from 565 nM to 10 μM, and the concentration of DA and VSP-77 from 137 nM to 100 μM. Concentration is represented as log_10_ scale (*n* = 3, error bars = SEM).

With this synthetic compound in hand, we first defined the agonism of VSP-77 in Cos-7 cells by using a PPARγ-activated luciferase reporter assay, in which Rosi and DA were used as the reference compounds. As expected and shown in Fig. 3a, VSP-77 has only a weakly agonistic activities at 33 μM and even at 100 μM. The dose–response curve in Fig. 3b suggests that VSP-77 has a potency similar to that of the partial agonist DA, and much lower than that of the full agonist Rosi.

**Fig. 4:**
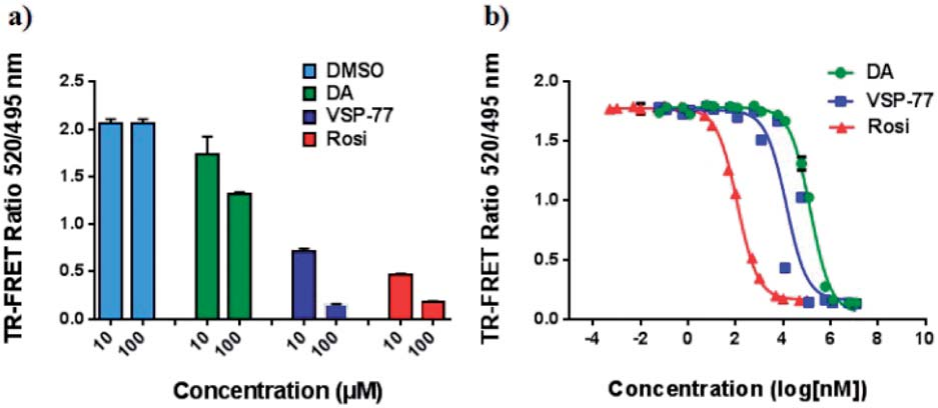
Characterization of VSP-77 binding. LanthaScreen™ TR-FRET assay (Invitrogen) was used for measuring the binding affinities of the ligands. 0.5 nM GST-PPARγ LBD was incubated with 5 nM Fluormone™ and Terbium-coated GST antibody. Increasing concentrations of DA, VSP-77 and Rosi competed the interaction, resulting in a decrease in the FRET ratio. (a) Competition by 10 μM and 100 μM of DA, VSP-77 and Rosi, respectively. (b) Dose–response competition curves for DA and VSP-77 from 1.2 × 10^7^ to 1.2 × 10^–2^ nM, and for Rosi from 1 × 10^5^ to 1 × 10^–3^ nM. Concentration is represented on a log_10_ scale. The calculated *k*_i_ values of DA, VSP-77 and Rosi are 50.5 μM, 4.8 μM and 42.8 nM, respectively (*n* = 3, error bars = SEM).

To determine the binding affinity of VSP-77 to PPARγ, we performed competition experiments using LanthaScreen™ TR-FRET assays. Fig. 4a and b show the competition of an indirectly labeled pan-PPAR ligand, Fluormone™, by VSP-77, as well as by DA and Rosi as positive controls. As shown in Fig. 4b, VSP-77 competed Fluormone about ten times more potently (*k*_i_ = 4.8 μM) than DA (*k*_i_ = 50.5 μM). Moreover, the saturation levels obtained with VSP-77 were similar to that of Rosi. These results confirm our structure-based design and also provide a basis for further evaluation of VSP-77 as a candidate compound for potent treatment of T2DM.

### VSP-77 demonstrated the specific selectivity towards PPARγ

**Fig. 5:**
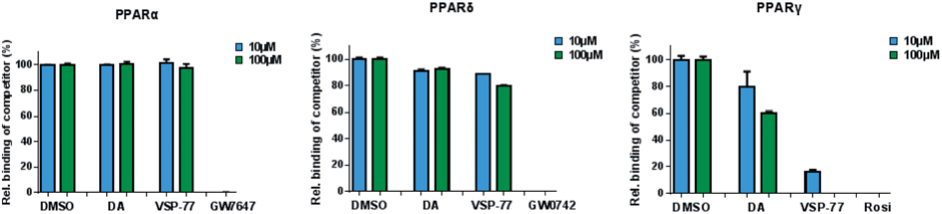
Identification of VSP-77 as a selective agonist toward PPARγ. Competitive TR-FRET assays using the indicated receptors. The positive controls for PPARα, PPARδ and PPARγ agonists are GW7647, GW0742 and Rosi, respectively (*n* = 3, error bars = SEM).

The PPAR family has three members in humans: PPARα, PPARδ and PPARγ.^50^,^51^ To validate the selectivity of VSP-77, we carried out competitive TR-FRET assays using the parent compound DA as reference and the well-defined and highly selective PPARα agonist GW7647, PPARδ agonist GW0742 and PPARγ agonist Rosi as positive controls. As shown in Fig. 5, GW7647, GW0742 and Rosi showed very strong binding capabilities at 10 μM (nearly 100%) for PPARα, PPARδ and PPARγ, respectively, which was in line with the previous reports.^9^,^52^,^53^ Interestingly, VSP-77 at 10 μM displayed a potent binding affinity with PPARγ, but had no obvious effects on PPARα and PPARδ even at 100 μM concentration. The results revealed that VSP-77 has an excellent selectivity toward PPARγ for its activation.

### VSP-77 only marginally stimulates adipocyte differentiation and induces the expression of key adipogenic genes

**Fig. 6:**
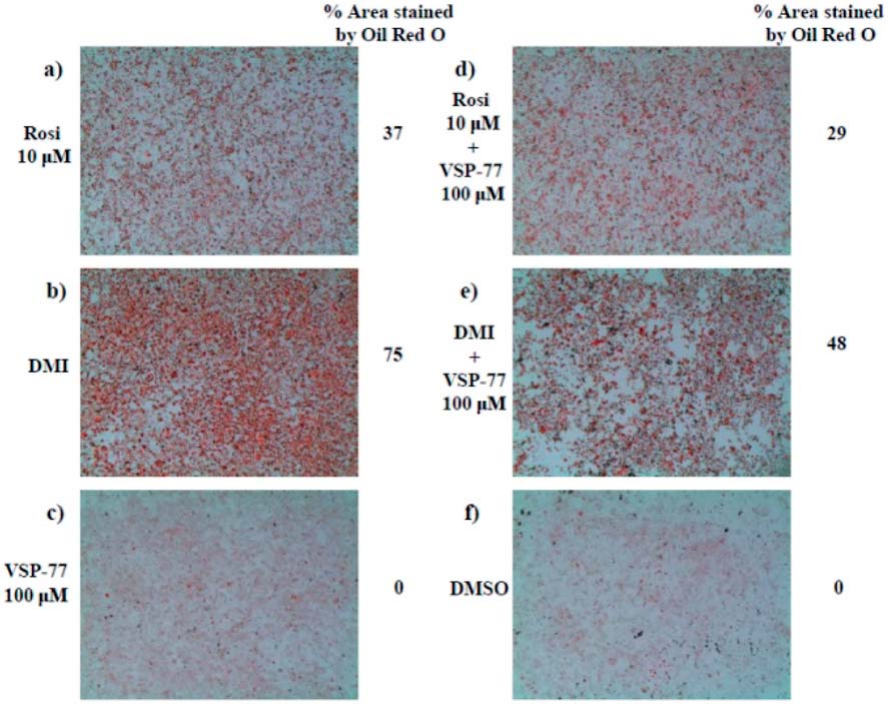
3T3-L1 adipocyte differentiation assay. Oil Red O staining of 3T3-L1 fibroblast cells after differentiating with different ligands for 2 days. Treatment groups include the following: (a) Rosi 10 μM; (b) DMI [1 μM dexamethasone (D); 0.5 mM 3-isobutyl-1-methylxanthine (M); and 167 nM insulin (I)]; (c) VSP-77 100 μM; (d) Rosi 10 μM + VSP-77 100 μM; (e) DMI + VSP-77 100 μM; (f) DMSO control.

One of the best documented side-effects of the PPARγ agonist drugs is weight gain as PPARγ is the key activator of adipogenesis. To determine whether VSP-77 promotes adipogenesis, we treated mouse fibroblast 3T3-L1 cells with 100 μM of VSP-77. Cells were also separately treated with Rosi (10 μM) or DMI (dexamethasone 1 μM, 3-isobutyl-1-methylxanthine 0.5 mM and insulin 167 nM, a standard inducer of adipocyte differentiation). Cells were incubated with Oil Red O, which stains adipocytes red. As shown in Fig. 6, adipocyte differentiation was observed after the treatment with Rosi and DMI, as evidenced by Oil Red O staining of the cellular lipid (Fig. 6a and b). In contrast, when incubated with 100 μM of VSP-77, cells did not stain with Oil-red O (Fig. 6c). Therefore, VSP-77 did not promote any detectable increase in lipid accumulation or changes in morphology characteristic of differentiating fat cells (Fig. 6c and f). Moreover, when VSP-77 was added to DMI- or Rosi-treated cells, we observed a clear decrease in the total number of adipocytes (Fig. 6d and e). Collectively, we conclude that VSP-77 at 100 μM dose does not activate adipogenesis despite partially activating PPARγ, and furthermore that VSP-77 can inhibit adipogenesis activated by DMI and Rosi. These results indicate that VSP-77 may function as a SPPARγM with decreased side effect on weight gain compared with Rosi.

**Fig. 7:**
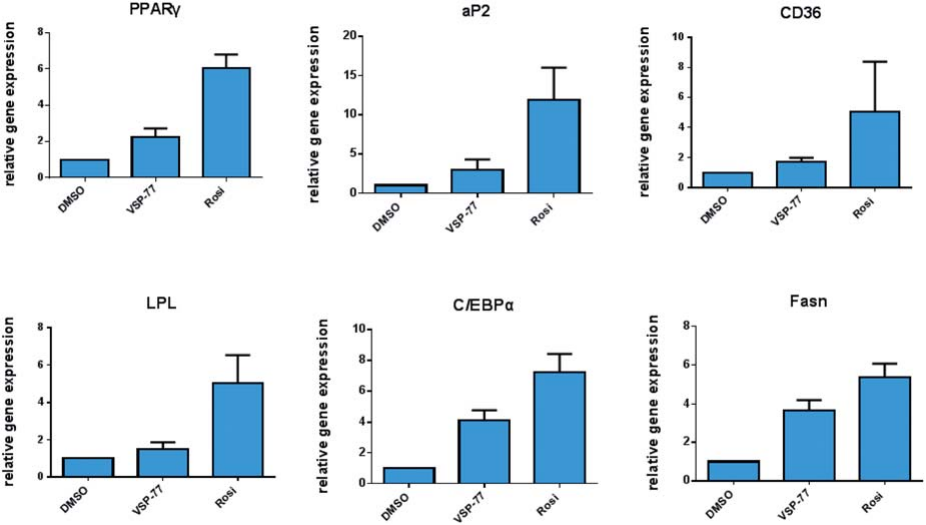
Expression of adipogenic genes in 3T3-L1 cells was analyzed by quantitative PCR (qPCR). Relative mRNA levels of the adipocyte differentiation genes PPARγ, aP2, CD36, LPL, C/EBPα and Fasn. mRNA was extracted from differentiating cells 7 days after treatment (*n* = 3, error bars = SEM).

Encouraged by the above results and to further determine whether VSP-77 activates PPARγ in 3T3-L1 cells, we evaluated the ability of VSP-77 to stimulate PPARγ activity by measuring the mRNA levels of endogenous PPARγ-regulated downstream genes linked to adipogenesis (primers are listed in Table S1†). Quantitative PCR was performed on 3T3-L1 cells 7 days after treatment with either VSP-77 or Rosi. As revealed in Fig. 7, Rosi robustly induced the expression of key adipogenic genes encoding PPARγ, aP2, CD36, LPL, C/EBPα and Fasn. In contrast, VSP-77 only weakly stimulated the expression of these target genes. These results suggest VSP-77 would not induce adipocytes differentiation through PPARγ, which is in agreement with VSP-77 being a relatively safe SPPARγM with decreased side effect on weight gain.

### Anti-diabetic activity of VSP-77 in high-fat diet (HFD) mice

Given the above data, we then examined whether VSP-77 had anti-diabetic properties *in vivo*. Normal mice become obese and insulin resistant following high fat-diet (HFD) fed,^54^,^55^ with activation of protein kinase CDK5 in their adipose tissues.^56^,^57^ Briefly, HFD mice was injected with VSP-77 for 10 days, and VSP-77 caused a decrease in the Cdk5-mediated phosphorylation of PPARγ at Ser-273 in adipose tissue, which was similar to the effects of Rosi administration. But unlikely Rosi administration, VSP-77 selectively induced the expression of glut4 and adiponectin, which was good for the improvement of insulin sensitivity (Fig. S1†). Moreover, VSP-77 treatment also improved the glucose tolerance, lowered glucose levels, and a significant reduction in the fasting insulin levels. Insulin resistance, as computed by HOMA-IR, showed a clear and dose-dependent improvement with VSP-77. These changes occurred without significant differences in body weight and food intake compared to vehicle-treated mice (Fig. S2†).

### VSP-77 has a good pharmacokinetic profile

VSP-77 was further evaluated for its preliminary pharmacokinetic profile in mice following oral (10 mg kg^–1^) and intravenous (5 mg kg^–1^) administration (Table S2†). The results revealed that VSP-77 given orally at 10 mg kg^–1^ dosage displayed a *T*_max_ of 0.5 h, a *C*_max_ of 2923 ng mL^–1^, an AUC_0–*t*_ of 5772 ng mL^–1^ h^–1^, an AUC_0–∞_ of 5877 ng mL^–1^ h^–1^, a mean retention time (MRT) of 1.94 h, a *t*_1/2_ of 1.25 h, and a 57.7% oral bioavailability, revealing that VSP-77 had a good pharmacokinetic profile.

### VSP-77 lacks detectable hERG inhibitory activity

In view of excellent biological activities of VSP-77 exhibited both *in vitro* and *in vivo*, we were interested to evaluate its hERG potassium ion channel inhibition profile. hERG inhibition is a common side effect of many drugs, including Rosi,^58^ and therefore an important anti-target for drug development. As listed in Table S3,† the reference marketed drug cisapride had a remarkable hERG potassium ion channel inhibitory activity with an IC_50_ value of 0.09 μM. In contrast, VSP-77 showed no obvious inhibition of hERG potassium ion channel even at 50 μM.

### VSP-77 has a unique PPARγ binding mode

**Fig. 8:**
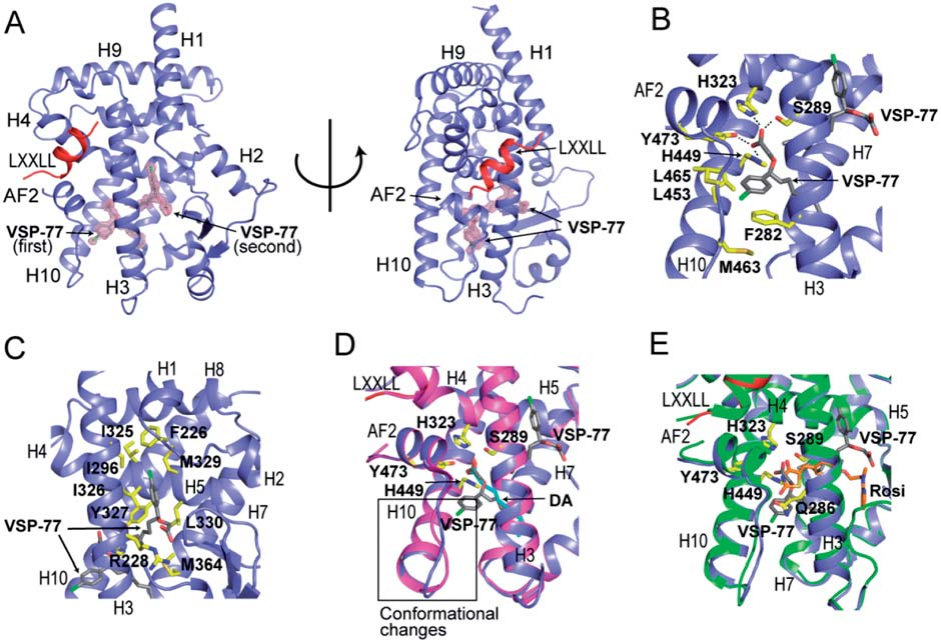
Crystal structure of PPARγ LBD in complex with (S)-VSP-77 and an LXXLL motif from PGC1, in which PPARγ isoform 1 was used for this study. (A) The overall complex crystal structure in two views. PPARγ LBD is colored in blue, the LXXLL motif in red, and the two molecules of (S)-VSP-77 are colored in gray and covered with a 2*F*_o_–*F*_c_ model map contoured to one sigma. (B) Binding mode of the first (S)-VSP-77 molecule. (S)-VSP-77 is colored in gray, and the protein residues interacting with (S)-VSP-77 are shown in yellow stick representation. The hydrogen bonds between the carboxyl group of the ligand and receptor residues are indicated by dashed lines. (C) Binding mode of the second (S)-VSP-77 molecule; same color code as that in panel B. (D) Superposition of (S)-VSP-77-bound (blue) with DA-bound (magenta) PPARγ LBD structures. (S)-VSP-77 is colored in gray, and DA is colored in cyan. The extra chlorophenyl group of (S)-VSP-77 causes conformational differences in the C-terminus of helix 10 and the following loop of the PPARγ LBD compared to those of DA-bound receptor. (E) Superposition of (S)-VSP-77-bound (blue) with Rosi-bound (green) PPARγ LBD structures. (S)-VSP-77 is colored in gray, and Rosi is in brown.

Encouraged by the aforementioned results and to investigate the molecular basis of how VSP-77 specifically regulates PPARγ activation, we determined the crystal structure of PPARγ LBD in complex with (S)-VSP-77 and a peptide encompassing the LXXLL motif from the PPARγ coactivator PGC1 by X-ray crystallography to a resolution of 1.43 Å (PDB code: 6MS7, Fig. 8A). The detailed statistics of diffraction data collection and structure refinement are summarized in Table S4.† As demonstrated in Fig. 8A, the (S)-VSP-77-bound PPARγ LBD adopts an active conformation with its C-terminal activation function-2 helix (AF-2) packed closely with helices 3 and 4 of the LBD, forming a coactivator binding site, where the LXXLL coactivator motif is docked. The conformation of the (S)-VSP-77-bound PPARγ LBD resembles the DA- and Rosi-bound LBD structures (PDB codes ; 3U9Q (ref. 46) and ; 3CS8 (ref. 59), respectively), with a large ligand binding pocket of about 1500 Å located in the lower part of the LBD.^3^

We designed VSP-77 based on the binding mode of DA to PPARγ LBD, with an oxygen atom replacing the β-carbon and a chlorophenyl moiety attached to the γ-position of DA (Fig. 1). We expected that the main chain of VSP-77 would occupy the same position as DA in the PPARγ ligand binding pocket, with the chlorophenyl group filling the portion of the ligand binding pocket near the γ-position of DA. Indeed, we found one (S)-VSP-77 molecule in the ligand binding pocket at the position as we expected. To our surprise, we found a second (S)-VSP-77 molecule in the PPARγ ligand binding pocket, filling the upper portion of the pocket, which was unoccupied in either the DA- or Rosi-bound PPARγ LBD structure (Fig. 8A–C).

The first (S)-VSP-77 molecule is located in the lower portion of the ligand binding pocket of PPARγ with its carboxyl group and backbone nicely overlaid with DA (Fig. 8A and D). The carboxyl group of this compound binds to PPARγ in the same mode as that of DA, forming a hydrogen bond network with the side chains of S289 of helix 3, H323 of helix 4, H449 of helix 10 and Y473 of the AF2 helix of the receptor. The chlorophenyl moiety of the compound occupies the portion of the ligand binding pocket surrounded by helices 3, 10 and AF2, and the loop connecting helices 10 and AF2, which is contacted in both DA- and Rosi-bound PPARγ structures (Fig. 8B). The phenyl part of the chlorophenyl moiety forms Van-der-Waals interactions with residues F282 of helix 3, L453 of helix 10, L469 and Y473 of AF2, M463 and L465 on the loop between helix 10 and AF2 helix, and the backbone of Q286 of helix 3, of the receptor. The chlorine atom of this chlorophenyl moiety points to the loop preceding the AF2 helix and forms polar interactions with the carbonyl group of S464 of this loop, and the side chain of Q286 of helix 3 (Fig. 8B). Obviously, the whole binding interface between (S)-VSP-77 and PPARγ LBD differs from that between DA and the receptor LBD due to the extra chlorophenyl moiety. The binding of this moiety in the PPARγ ligand binding pocket may be related to the conformational differences in the C-terminal end of helix 10 and the following loop of the PPARγ LBD (Fig. 8D), and is likely a main contribution to the enhanced binding affinity to the receptor (Fig. 3 and 4).

The second (S)-VSP-77 molecule in the ligand binding pocket of the receptor occupies the upper portion of the large ligand binding pocket, which is not observed in the DA-bound complex (Fig. 8A, C and D). The chlorophenyl moiety of the ligand is in a highly hydrophobic region in the upper portion of the pocket, which was unoccupied in the DA- and Rosi-bound structures. It is surrounded by helices 3 and 7 and the loop connecting helices 1 and 2, forming hydrophobic interactions with residues F226 on the loop following helix 1, A292 and I296 of helix 3, and I325, I 326 and M329 of helix 7 of the receptor LBD (Fig. 8C). The hydrophobic tail of this molecule is at the central portion of the ligand binding pocket, and surrounded by residues I326, Y327 and L330 of helix 4, M364 of helix 7, H449 of helix 10, of the receptor, as well as the backbone of the first (S)-VSP-77 molecule. The carboxyl group of the second (S)-VSP-77 molecule points out from the pocket through the cleft between helix 3 and the β-sheet. It forms a salt bridge with R228 of helix 3, whose side chain is flipped about 180 degree from its positions in DA- and Rosi-bound receptor to facilitate a close interaction with the carboxyl group of (S)-VSP-77 (Fig. 8C). The portion of the ligand binding pocket occupied by the second (S)-VSP-77 molecule is largely unoccupied in both DA- and Rosi-bound PPARγ LBD, which likely contributes to the specific agonist activity of this compound to PPARγ activation.

The binding mode of (S)-VSP-77 is largely different from that of Rosi (Fig. 8E). The TZD head of Rosi can be partially overlaid with the carboxyl group of the first (S)-VSP-77 molecule, but binds in a different mode to PPARγ residues. Specifically, the carboxyl group of (S)-VSP-77 binds to receptor residues S289 of helix 3, H323 of helix 4, H449 of helix 10, and Y473 of AF2, while the TZD group of Rosi binds to residues Q286 and S289 of helix 3, H323 of helix 4, and Y473 of AF2, of the receptor. The different binding modes are mainly due to the different chemical structures and orientations of these two groups in the ligand binding pocket. The backbone of Rosi is located in the central portion of the large ligand binding pocket of PPARγ LBD, partially overlapping the alkyl tail of the second (S)-VSP-77 molecule. Its methylamino-pyridine tail occupies the lower portion of the ligand binding pocket between helix 3 and the small β-sheet of the LBD, which is unoccupied in both DA- and (S)-VSP-77-bound PPARγ LBD structures (Fig. 8D and E). While all of the above binding specificities may associate with the different activities between (S)-VSP-77 and Rosi, the binding of the compounds to residues close to the co-activator binding site may play more important roles in their distinct PPARγ activations.

### (S)-VSP-77 is the potential conformation that has potent anti-diabetic activity and does not promote fluid retention, weight gain and haemodilution in HFD mice

**Fig. 9:**
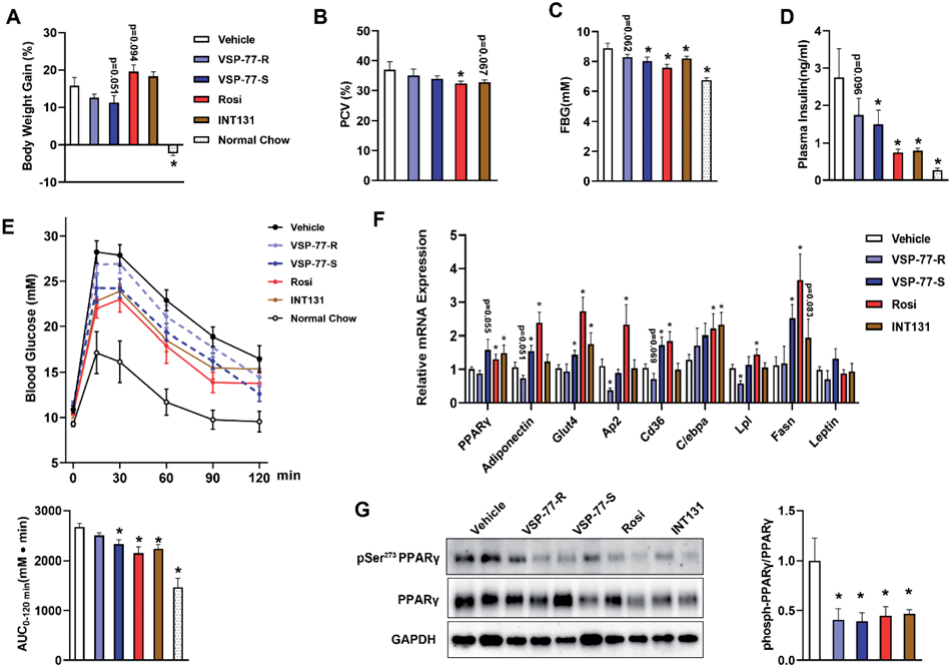
(S)-VSP-77 has potent anti-diabetic activity and does not promote fluid retention in ob/ob mice. Mice were intraperitoneally injected with vehicle, (R)-VSP-77 (5 mg kg^–1^), (S)-VSP-77 (5 mg kg^–1^), Rosi (10 mg kg^–1^), or orally treated with INT131 (30 mg kg^–1^) for 7 weeks. (A) Body weight gain. (B) Packed cell volume (PCV) in whole blood. (C) Fasting blood glucose. (D) Fasting blood insulin. (E) Blood glucose levels after an intraperitoneal glucose load (2 g kg^–1^) performed after 6 week treatment. The areas under the curve are indicators of glucose clearance. (F) Relative mRNA expression. (G). The level of Ser-273 phosphorylation of PPARγ in white adipose tissue by using PPARγ isoform 2. *P < 0.05 compared with vehicle (*n* = 6–7, error bars = SEM).

Inspired by the above structural information, we further synthesized and separated (S)-VSP-77 and (R)-VSP-77 (see the ESI† for details) to verify the potential conformation for presenting the anti-diabetic effects in HFD mice. Herein, INT131, a classical PPARγ partial agonist, was selected as the positive control. Weight gain and fluid retention caused by TZD drugs like rosiglitazone are suspected to be key factors in their increased cardiac risk. HFD mice were injected with (S)- or (R)-VSP-77 for 7 weeks. As shown Fig. 9A and B, either (S)-VSP-77 or (S)-VSP-77 treatment did not induce body weight gain and haemodilution, while these changes were observed in Rosi-treated mice. (S)-VSP-77 treatment but not (R)-VSP-77 caused a significant reduction in fasting glucose and insulin levels (Fig. 9C and D). As predicted, (S)-VSP-77 treated mice exhibited better blood glucose clearance than the control mice, which was similar to high-dose Rosi-treated and INT131-treated mice (Fig. 9E).

We further characterized the ability of (S)-VSP-77 to stimulate PPARγ activity by measuring the *in vivo* mRNA levels of endogenous PPARγ-regulated downstream genes linked to adipogenesis and insulin sensitivity. Quantitative PCR was performed on adipose tissue. As illustrated in Fig. 9F, Rosi robustly stimulated the expression of key adipogenic genes encoding PPARγ, AP2, CD36, LPL, C/EBPa and Fasn. In contrast, (S)-VSP-77 weakly induced the expression of these genes than Rosi, which was in good agreement with our observation in adipogenesis. Of note, Glut4 and Adiponectin that contributed to insulin sensitivity were increased in both (S)-VSP-77 and Rosi-treated mice. In addition, we also investigated whether (S)-VSP-77 exerts this biochemical function in white adipose tissue from each treatment group. As shown in Fig. 9G, (S)-VSP-77 effectively blocked CDK5-mediated Ser-273 phosphorylation, which was similar to Rosi and INT131. In combination with the above results, we concluded that (S)-VSP-77 is the potential conformation and may exert an anti-diabetic effect through the modulation of the expression of the insulin sensitivity-related genes Glut4 and Adiponectin and the inhibition of CDK5-mediated PPARγ-Ser-273 phosphorylation.

## Discussion

Since the treatment of T2DM with full PPARγ agonists including TZD drugs is associated with many severe side effects, the development of new classes of alternative PPARγ ligands with partial agonism has received intensive research focus in modern medicinal chemistry. In view of these advances, SPPARγMs were found to occupy a dominant role in this important research area. They are thought to selectively recruit co-activators and activate PPARγ, thereby maintaining therapeutic benefits with minimized side effects. However, up to date, no SPPARγMs have been successfully applied in clinical practice for the treatment of T2DMs and mechanistically it remains unclear how to achieve selective PPARγ activation. In this paper, we employed a structure-based design and then identified a novel synthetic “hit” compound, VSP-77, which acts as a selective activator of PPARγ without activation of either PPARα or PPARδ. The results from both the biochemical LanthaScreen TR-FRET assay and the cell-based reporter gene assay demonstrate that VSP-77 is a selectively modulating ligand for PPARγ with partial agonism. Compared with its parent compound DA, VSP-77 exhibits a potent binding affinity for PPARγ (about 10-fold higher than DA) with excellent selectivity towards PPARγ.

VSP-77 has several key features that distinguish it from full agonist TZDs. First, the scaffold of VSP-77 is distinct from that of TZDs. The carboxyl group of VSP-77 forms alternative hydrogen bond interactions compared to the TZD moiety of TZD drugs, and VSP-77 utilizes both alkyl chain and phenyl group as the backbone to form possible lipophilic interactions. Second, transcriptional activation of PPARγ induces adipocyte differentiation, and PPARγ full agonists, such as TZDs, have strong adipogenic capacity, which is one of the major factors leading to their undesirable side effects. In sharp contrast, VSP-77 has almost no adipogenic activity despite partially activating PPARγ, thus revealing that VSP-77 has obvious advantages over marketed TZD drugs. This conclusion is further supported by the subsequent quantitative PCR analysis of PPARγ-regulated key adipogenic genes including PPARγ, aP2, CD36, LPL, C/EBPα and Fasn. Moreover, the preliminary anti-diabetic effect of VSP-77 has been confirmed in the short-term HFD mouse model (each group treated for 10 days). Compared to the multi-billion dollar TZD drug Rosi, our studies in mice demonstrate that VSP-77 has clear therapeutic benefits. For example, VSP-77 displays a significantly better blood glucose clearance than Rosi. Furthermore, VSP-77-treated mice selectively decreased subcutaneous and perirenal fats, while Rosi-treated mice clearly increased liver weight. Further pharmacokinetics studies indicate that VSP-77 has a highly attractive pharmacokinetic profile, such as good exposure (an AUC_0–*t*_: 5772 ng mL^–1^ h^–1^, an AUC_0–∞_: 5877 ng mL^–1^ h^–1^) and oral bioavailability (*F* = 57.7%). Finally, VSP-77 treatment shows no detectable hERG inhibition even at 50 μM. In sharp contrast, Rosi has a relatively high inhibitory activity with an IC_50_ value of 18.8 μM.^58^

To gain further insight into the anti-diabetic mechanism of VSP-77, we also performed quantitative PCR analysis on adipose tissue to measure the mRNA levels of endogenous PPARγ-regulated downstream genes linked to adipogenesis and insulin sensitivity. The results show that VSP-77 does not induce the expression of key adipogenesis-related genes, yet selectively activates the expression of Glut4 and Adiponectin, which contribute to insulin sensitivity. These results are in good agreement with our above obtained conclusion *in vitro* that VSP-77 has an improved therapeutic profile over TZDs. Moreover, It was known that Cdk5-mediated phosphorylation of PPARγ at Ser-273 was obesity-linked phosphorylation site. Recently developed several partial agonists like SR1664 and MRL24 clearly blocked the obesity-linked phosphorylation site to present the improved therapeutic index for anti-diabetic drug discovery.^57^ Indeed, the correlation between inhibition of this phosphorylation and the therapeutic effects *in vivo* suggested that it might be possible to create novel SPPARγMs which are effective for T2DM with fewer side effects.^19^ Consistently, we found that VSP-77 effectively reduced the Ser-273 phosphorylation level of PPARγ in a CDK5-dependent signalling assay. Taken together, we have provided clear evidence that VSP-77 is a partial agonist for PPARγ with therapeutic advantages over Rosi that functions as a PPARγ-modulating ligand.

Subsequently our high resolution crystal structure of PPARγ LBD in complex with two (S)-VSP-77 molecules has demonstrated an extended binding interface and novel binding mode between (S)-VSP-77 and the receptor that is occupied by the introduced chlorophenyl group at the γ-position of DA. In the structure, the carboxyl head group of the first (S)-VSP-77 molecule perfectly overlays with the carboxyl group as DA. Consistently, it forms a hydrogen bond network with ligand binding pocket residues to stabilize the active conformation of the PPARγ LBD in the same mode as DA. However, the addition of a chlorophenyl group to the backbone makes (S)-VSP-77 better fit in the hydrophobic ligand binding pocket, and increases the binding affinity to the receptor. In addition, we completely unexpectedly found a second (S)-VSP-77 molecule to occupy the upper portion of the PPARγ ligand binding pocket, which was unoccupied in both the DA- or Rosi-bound PPARγ LBD structures.

Finally, we further investigated the potential conformation of racemate VSP-77 in the long-term HFD mice (each group treated for 7 weeks). The results revealed that (S)-VSP-77 is the potential conformation, and it exhibits potent anti-diabetic activity through the modulation of the expression of the insulin sensitivity-related genes Glut4 and Adiponectin and the inhibition of CDK5-mediated PPARγ-Ser-273 phosphorylation, which is in line with our observation from both *in vitro* and *in vivo* studies and the co-crystal structural analysis. Importantly, it does not promote fluid retention, weight gain as well as haemodilution in the current investigation, indicating that (S)-VSP-77 has a relatively good safe profile for clinical use. Besides, our data also showed that low-dose (S)-VSP-77 treated mice exhibited effective anti-diabetic effects that was similar to relatively high-dose INT131-treated mice (5 mg kg^–1^ for (S)-VSP-77 *vs.* 30 mg kg^–1^ for INT131). Taken together, these result presented in here not only revealed that (S)-VSP-77 has a more potent anti-diabetic effect than INT131 but also suggested that (S)-VSP-77 can serve as a promising candidate for the treatment of T2DM and as the lead compound for designing better and safer pharmacological agents by selectively targeting PPARγ.

## Conclusions

In summary, we here have identified the DA-based VSP-77 as a novel and versatile PPARγ ligand. Compared to the currently marketed anti-diabetic drug Rosi, VSP-77 has several remarkable features: (i) it is a selectively PPARγ-modulating ligand with partial agonism; (ii) it displays a potent binding affinity for PPARγ; (iii) it presents an excellent selectivity towards PPARγ; (iv) it does not stimulate adipocyte differentiation and does not or only marginally induce the expression of key fat cell genes, including PPARγ, aP2, CD36, LPL, C/EBPα and Fasn; (v) it has a good pharmacokinetic profile with excellent exposure and oral bioavailability; (vi) it exhibits a potent anti-diabetic effect without causing key tissue weight gain through selectively increasing the expression of Glut4 and Adiponectin and blocking the Cdk5-mediated phosphorylation of PPARγ at Ser-273; (vii) it does not show any inhibitory activity of hERG potassium ion channel under the high dosage condition; (viii) the co-crystal structure of PPARγ bound to two molecules of (S)-VSP-77 reveals a previously undisclosed allosteric binding mode. Subsequently, single chiral (S)-VSP-77 has been synthesized and separated as the potential conformation for the above proof-of-principle demonstration. Undoubtedly, clinical assays are necessary to unambiguously determine T2DM-related pharmacological and physiological actions to give a comprehensive view on the anti-diabetic efficiencies *vs.* adverse effects of (S)-VSP-77. Nevertheless, our findings have not only gave a clear evidence to support that, VSP-77, especially (S)-VSP-77, has good druggability and highly attractive advantages over the marketed TZD drug Rosi and representative partial agonist INT131, but also provided a new and rational basis for next stage of designing novel SPPARγMs as potent anti-diabetic drugs with improved therapeutic profile and minimized side effects.

## Ethical statement

All animal procedures were performed in accordance with the Guidelines for Care and Use of Laboratory Animals of Shanghai Institute of Materia Medica (SIMM), Chinese Academy of Sciences and approved by the Institutional Animal Care and Use Committee (IACUC) of SIMM (Note: SIMM is an institution with AAALAC (The Association for Assessment and Accreditation of Laboratory Animal Care) International Accreditation)

## Conflicts of interest

There are no conflicts to declare.

## Supplementary Material

Supplementary information
