# Supplementary material for: Identification and structural insight of an effective PPARγ modulator with improved therapeutic index for anti-diabetic drug discovery
Source: Chem Sci. 2020 Jan 21;11(8):2260–8. doi: 10.1039/c9sc05487a (PMC7059199; doi:10.1039/c9sc05487a)
Supplement: Supplementary file 1 [file SC-011-C9SC05487A-s001.pdf]

Electronic Supplementary Material (ESI) for Chemical Science.
This journal is © The Royal Society of Chemistry 2020

Supplementary Information

for

Identification and structural insight of an effective PPARγ modulator with improved therapeutic index for anti-diabetic drug discovery

Haowen Jiang,[‡ac] X. Edward Zhou,[‡d] Jingjing Shi,[‡b] Zhi Zhou,[‡a] Guanguan Zhao,[b] Xinwen Zhang,[c] Yili Sun,[c] Kelly Suino-Powell,[d] Lei Ma,[a] Hui Gao,[a] Xiyong Yu,[a] Jia Li,*[c] Jingya Li,*[c] Karsten Melcher,[d] H. Eric Xu*[abd] and Wei Yi*[ab]

[a]Guangzhou Municipal and Guangdong Provincial Key Laboratory of Protein Modification and Degradation & Molecular Target and Clinical Pharmacology, State Key Laboratory of Respiratory Disease, School of Pharmaceutical Sciences & the Fifth Affiliated Hospital, Guangzhou Medical University, Guangzhou, Guangdong 511436, China
[b]VARI/SIMM Center, Center for Structure and Function of Drug Targets, CAS-Key Laboratory of Receptor Research, Shanghai Institute of Materia Medica, Chinese Academy of Sciences, Shanghai 201203, China
[c]National Center for Drug Screening, State Key Laboratory of Drug Research, Shanghai Institute of Materia Medica, Chinese Academy of Sciences, Shanghai 201203, China
[d]Structural Biology Program, Center for Cancer and Cell Biology, Van Andel Research Institute, Grand Rapids, Michigan 49503, USA
[‡]These authors contributed equally.

*To whom correspondence should be addressed to W.Y. (yiwei@gzhmu.edu.cn), J.L (jyli@simm.ac.cn, jli@simm.ac.cn) or H.E.X (eric.xu@simm.ac.cn).

Table of Contents:

Supplementary Figure 1. Relative mRNA expression and the level of Ser-273 phosphorylation of PPARγ in white adipose tissue

Supplementary Figure 2. Antidiabetic effects of VSP-77 in high fat-diet mice.

Section 1. Experimental part

Section 2. References

Section 3. Copies of ^{1}H and ^{13}C NMR spectra of products 1-4 and VSP-77

Section 4. Design of DA-based PPARγ agonists (Figure S1)

Section 5. Primer sequences used for qPCR (Table S1)

Section 6. Pharmacokinetic parameters for VSP-77 (Table S2)

Section 7. Inhibition Assay of VSP-77 on the hERG Potassium Ion Channel (Table S3)

Section 8. Crystal data collection and structure refinement statistics (Table S4)

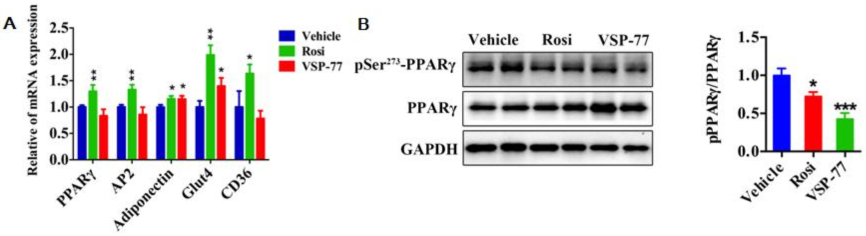

Fig S1. Relative mRNA expression (A) and the level of Ser-273 phosphorylation of PPARγ (B) in white adipose tissue. Mice were treated with VSP-77 or Rosi for 10 days in HFD mice. *P<0.05, **P<0.01 compared with vehicle (n = 5-7, error bars = SEM).

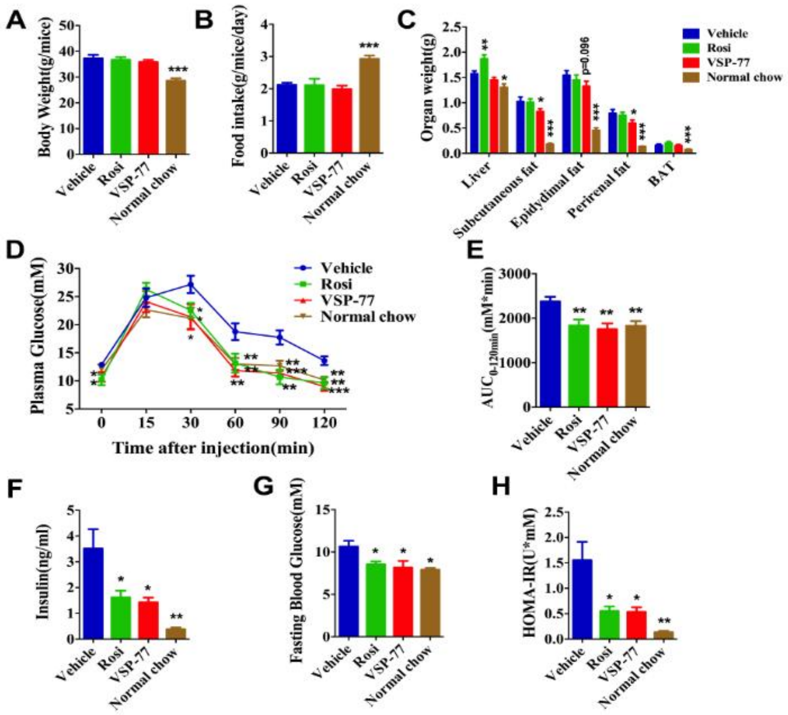

Fig S2. Antidiabetic effects of VSP-77 in high fat-diet mice. Mice were intraperitoneally injected with vehicle, VSP-77 (5 mg/kg) or Rosi (5 mg/kg), respectively. A: Body weight of day 10. B: Food intake. C. Organ weight. D-E: Blood glucose levels after an intraperitoneal glucose load (2 g/kg) performed after 10 days of treatment. The areas under the curve are indicators of glucose clearance. F. Basal blood insulin. G. Fasting blood glucose. H. HOMA-IR. *P<0.05, **P<0.01,***P<0.001 compared with vehicle (n = 5-6, error bars = SEM).

Section 1. Experimental part.

Procedure for the synthesis of VSP-77

(i) To a stirred solution of 4-chlorobenzaldehyde (1.40 g, 10 mmol) in dry THF (30 mL) at 0 ^{o}C, heptylmagnesium bromide (2.03 g, 10 mmol) was dropwise added and stirred at room temperature for 24 h in nitrogen. After the reaction was completed, a

saturated solution of ammonium chloride (20 mL) was added. Then the organic phase was separated, dried over anhydrous Na_2SO_4 and the solvent was removed under reduced pressure. The residue was purified by flash chromatography on silica gel (eluent: petroleum ether/ethyl acetate 5:1) to afford the desired product **1** as a yellow liquid (yield 85%). ^{1}H NMR (400 MHz, $CDCl_3$) δ 7.32 (d, J = 8.6 Hz, 2H, ArH), 7.27 (d, J = 8.6 Hz, 2H, ArH), 4.63 (t, J = 6.6 Hz, 1H, ArCH), 1.98-1.66 (m, 2H, CH_2), 1.37-1.26 (m, 10H, CH_2), 0.87 (t, J = 6.7 Hz, 3H, CH_3); ^{13}C NMR (100 MHz, $CDCl_3$) δ 143.5, 133.1, 128.6, 127.4, 74.0, 39.2, 31.9, 29.6, 29.3, 25.8, 22.7, 14.2.

(ii) Sodium hydride 60% in mineral oil (0.30 g, 7.5 mmol) was carefully added to a stirred solution of **1** (1.20 g, 5 mmol) in dry THF (30 mL) at 45 ^{o}C. After 4 h ethyl 2-bromoacetate (1.00 g, 6 mmol) in dry THF (10 mL) was dropwise added, and the mixture was stirred for 24 h. After the reaction was completed, crushed ice (40 g) was carefully added. Then the organic phase was separated, dried over anhydrous Na_2SO_4 and the solvent was removed under reduced pressure. The residue was purified by flash chromatography on silica gel (eluent: petroleum ether/ethyl acetate 30:1) to afford the desired product **2** as a yellow liquid (yield 45%). ^{1}H NMR (400 MHz, $CDCl_3$) δ 7.32 (d, J = 8.4 Hz, 2H, ArH), 7.24 (d, J = 8.4 Hz, 2H, ArH), 4.32 (t, J = 6.6 Hz, 1H, $OCHCH_2$), 4.17 (qd, J = 7.2, 1.9 Hz, 2H, CO_2CH_2), 3.98 (d, J = 16.3 Hz, 1H, OCH_2CO_2), 3.83 (d, J = 16.3 Hz, 1H, OCH_2CO_2), 1.93-1.84 (m, 1H, CH_2), 1.67-1.59 (m, 2H, CH_2), 1.44-1.36 (m, 1H, CH_2), 1.32-1.16 (m, 8H, CH_2), 1.25 (t, J = 7.2, 3H, CH_3), 0.85 (t, J = 6.6 Hz, 3H, CH_3); ^{13}C NMR (125 MHz, $CDCl_3$) δ 170.5, 140.2, 133.6, 128.8, 128.4, 82.4, 66.0, 60.9, 53.6, 38.1, 31.9, 29.6, 29.3, 25.8, 22.8, 14.3, 14.2.

(iii) The mixture of **2** (0.33 g, 1mmol) and lithium hydroxide hydrate (0.10 g, 2.5 mmol) in EtOH (2 mL) was magnetically stirred at room temperature for 2 h. After most solvent was evaporated, the residue was dissolved in water (20 mL), the pH was adjusted to 1.0 with 1M HCl solution and extracted with EtOAc (3×20 mL). The organic extracts were dried over anhydrous Na_2SO_4 and the solvent was removed under reduced pressure. The residue was purified by flash chromatography on silica gel (eluent: petroleum ether/ethyl acetate 5:1) to afford the desired product VSP-77 as a yellow liquid (yield 80%). ^{1}H NMR (400 MHz, $CDCl_3$) δ 7.34 (d, J = 8.3 Hz, 2H, ArH), 7.21 (d, J = 8.3 Hz, 2H, ArH), 4.33 (t, J = 6.7 Hz, 1H, ArCH), 4.01 (dd, J = 16.4 Hz, 4.0 Hz, 1H, $CHCO_2$), 3.91 (d, J = 16.4 Hz, 1H, $CHCO_2$), 1.95-1.84 (m, 1H, CH_2), 1.73-1.59 (m, 2H, CH_2), 1.42-1.32 (m, 1H, CH_2), 1.25 (s, 9H, CH_2), 0.86 (t, J =

6.8 Hz, 3H, CH_3); ^{13}C NMR (100 MHz, $CDCl_3$) δ 175.7, 139.6, 133.9, 128.9, 128.3, 82.7, 65.5, 37.9, 31.9, 29.5, 29.3, 25.7, 22.7, 14.2; HR-MS (ESI) calcd for 297.1257 ($[M-H]^-$), found 297.1264 ($[M-H]^-$).

Procedure for the synthesis of (S)-VSP-77 and (R)-VSP-77

(i) A solution of VSP-77 (1.2 g, 4 mmol), (S)-benzenemethanamine (0.49 g, 4 mmol), HOBT (0.57 g, 4.2 mmol), and EDCI (0.81 g, 4.2mmol) in dry DMSO (20 mL) were stirred in ice bath. After 10 min, the reaction mixture was stirred for 4 h at room temperature. The mixture was extracted by ethyl acetate, washed by water for three times. The solvent was removed under reduced pressure. The residue was purified by chromatography on silica gel with a solution of petroleum ether and ethyl acetate as eluent to give desired products 3 and 4. For product **3** (42%) : ^1H NMR (400 MHz, $CDCl_3$) δ 7.43-7.30 (m, 7H), 7.21 (d, *J* = 8.4 Hz, 2H), 6.85 (d, *J* = 8.1 Hz, 1H), 5.23-5.13 (m, 1H), 4.26-4.17 (m, 1H), 3.81 (dd, *J* = 40.7, 15.3 Hz, 2H), 1.86-1.83 (m, 1H), 1.69-1.61 (m, 1H), 1.53 (d, *J* = 6.9 Hz, 3H), 1.36-1.17 (m, 10H), 0.89 (t, *J* = 7.0 Hz, 3H). For product **4** (37%): ^1H NMR (400 MHz, $CDCl_3$) δ 7.42 – 7.29 (m, 7H), 7.17 (d, *J* = 8.4 Hz, 2H), 6.85 (d, *J* = 8.0 Hz, 1H), 5.23-5.13 (m, 1H), 4.29-4.21 (m, 1H), 3.82 (s, 2H), 1.92-1.80 (m, 1H), 1.70-1.64 (m, 1H), 1.56 (d, *J* = 6.9 Hz, 3H), 1.36-1.17 (m, 10H), 0.89 (t, *J* = 7.0 Hz, 3H).

(ii) A solution of **3** or **4** (600 mg) in 35 mL of 6N HCl and 35 mL of Dioxane (1:1 mixture) was heated at 100 ℃ for 6 h. Being guided by TLC, reaction was stopped. Reaction mixture was diluted ethyl acetate (300 mL) and washed with water (200 mL).Organic layer was dried, condensed and the residue was purified by chromatography on silica gel with a solution of petroleum ether and ethyl acetate as eluent to give (S)-VSP-77 with 87% yield (395 mg) and (R)-VSP-77 with 81% yield (366 mg), respectively. The characterization data of ^1H NMR and ^{13}C NMR spectra is consistent with VSP-77.

Protein preparation

The LBD of PPARγ (codons 206-477) was cloned into the pSUMO vector (LifeSensors) with an N-terminal 6×His SUMO tag. The protein was expressed in *E. coli* BL21 (DE3) in LB broth at 25 ℃ to an A_{600} of 1.0 and induced with 0.1 mM of isopropyl 1-thio-β-d-galactopyranoside at 16 ℃. Cells were harvested, resuspended in 200 ml of extract buffer (50 mM of Tris (pH 8.0), 150 mM of NaCl, 10% of glycerol,

and 25 mM of imidazole) per 6 liters of cells, and passed three times through a French press with pressure set at 1000 Pa. The lysate was cleared by centrifugation at 48,000 x g for 30 minutes and the supernatant was purified by loading on 2x5 mL HiLoad Nickel HP columns (GE Healthcare). The 6 ×His SUMO tag was removed by cleavage with the Sumo protease Ulp1 at a protease: protein ratio of 1:1000), and the tag was separated from the PPARγ LBD by a second pass through the Nickel column. PPARγ monomer was further purified by gel filtration (HiLoad 26/60 Superdex 200 (GE Healthcare)) with 20 mM of Tris (pH 8.0), 100 mM of NaCl, and 5 mM of DTT as buffer. The PPARγ LBD protein was complexed with PGC1α1 peptide (AEEPSLLKKLLLAPA) at a 1:1.2 molar ratio and with compound at a 1:5 molar ratio and then filter-concentrated to 10 mg/mL.

Cell-based transactivation assay

Cos-7 cells from ATCC were grown to 70% confluence in DMEM containing 10% fetal bovine serum (FBS). For assessing full-length PPAR receptors, Cos-7 cells were transiently co-transfected with a plasmid containing the luciferase gene under the control of three tandem PPAR response elements (100 ng) (PPRE × 3 TK-luciferase) and 50 ng of full-length hPPARγ plasmids using lipofectamine 2000 (Invitrogen) along with the standard 10 ng of renilla luciferase gene. 4 hrs after transfection the cells were treated with the corresponding concentration of rosiglitazone, DA, and VSP-77 for 24 hours. Reporter luciferase assay kits from Promega were used to measure the luciferase activity, according to the manufacturer's instructions, with a luminometer (Envision, Perkin–Elmer). Luciferase activity was normalized to renilla units. Each condition was performed with $n \geq 3$ for each experiment. DMSO was used as control.

Competitive TR -FRET binding assay

The GST PPARγ -LBD was labeled with a terbium-linked antibody and a fluorescent small molecule pan-PPAR ligand (Fluormone™ Pan-PPAR Green, Invitrogen) was used as a tracer that is displaced from the ligand binding domain upon agonist binding. Excitation of the terbium at 340 nm results in FRET to the fluorescent tracer, with emissions detected at 520 nm and 495 nm. Tracer displacment results is a loss of FRET signal between the terbium label and the fluorescent tracer.

The inhibition constant (k_i) for a competitor was calculated by applying the

Cheng-Prusoff equation as following:

$$k_i = IC_{50} / \{ 1 + [tracer]/k_d \}$$

where IC_{50} is the concentration of competitor that produces 50% displacement of the tracer, [tracer] is the concentration of Fluormone™ Pan-PPAR Green used in the assay (5 nM), and KD is the binding constant of Fluormone™ Pan-PPAR Green to PPAR γ-LBD. This KD value has been determined to be 2.8 ± 0.8 nM (average ±standard deviation calculated from three separate experiments).

Adipocyte differentiation assay

The adipocyte differentiation assay was performed with NIH 3T3-L1 preadipocytes obtained from ATCC. The 3T3-L1 preadipocytes were maintained in DMEM containing 10% FBS and antibiotics. For differentiation DMI (Dexamethazone 1 μM, 3-isobutyl-1-methyxanthine (IBMX) 0.5 mM and Insulin167 nM) and 10 μM of rosiglitazone were used as positive controls for the assay. VSP-77 was used at a 100 μM concentration. All the treatments have insulin at 167 nM concentration. Media with DMSO was used as a negative control. Differentiation was induced by treating post-confluent cultures with media containing the respective ligands for two days. Cells were stained with Oil Red O to estimate the lipid accumulation. The images were analyzed using ImageJ (National Institutes of Health).

Gene expression analysis

For real-time PCR analysis, 1-2 μg total RNA was reverse-transcribed using the SuperScript cDNA Reverse Transcription Kit (Invitrogen) with SYBR Green PCR Master Mix (Invitrogen) and gene specific primers (Table S1) using a Roche Light Cycler 480 machine. The relative expression of mRNA was determined after normalization to β-actin level using the ΔΔ-Ct method.

Animal experiments

All animal procedures were performed in accordance with the Guidelines for Care and Use of Laboratory Animals of Shanghai Institute of Materia Medica (SIMM), Chinese Academy of Sciences and approved by the Institutional Animal Care and Use Committee (IACUC) of SIMM (Note: SIMM is an institution with AAALAC (The Association for Assessment and Accreditation of Laboratory Animal Care) International accreditation). Six-week old male C57BL/6J mice (Shanghai SLAC

Laboratory Animal Co., Shanghai, China) were housed in a temperature-controlled room (22 ± 2 ℃) with a light/dark cycle of 12 h. For chronic treatment, mice were fed high-fat diets (60% calories from fat; Research Diets, New Brunswick, NJ, USA) or standard diets ad libitum. At 14 weeks of age, mice were randomly assigned to treatment groups. For the study of effect on HFD mice, vehicle (PBS), rosiglitazone (5 mg/day/kg or 10 mg/kg/day), VSP-77 (5 mg/kg/day) and (S)- or (R)-VSP-77 (5 mg/kg/day) were intraperitoneally injected and INT131 (30 mg/kg/day) was orally treated. The glucose tolerance test (2 g/kg glucose i.p.) and determination of fasting blood glucose and insulin levels were performed after 6 h of starvation. At the end, the animals were anatomized and blood and tissues were collected for further investigation.

Crystallization, data collection and structure determination

The PPARγ/PGC-1α/VSP-77 crystals were grown at 25 ℃ in sitting drops containing 0.1 μL of the protein complex and 0.1 μL of well solution containing 0.1 M of tri-sodium citrate (pH 5.5), 20% w/v PEG 3350. Crystals were directly frozen in liquid nitrogen for data collection. The crystals formed in the $C222_1$ space group (Table S4). The datasets were collected with a MAR225 CCD detector at the ID line of sector 21 of the Advanced Photon Source at Argonne National Laboratory (Argonne, IL). The data was indexed and scaled with HKL2000 package[1] to 1.43 Å. The CCP4 program PHASER was used for molecular replacement (http://www.ccp4.ac.uk), with the PPARγ-Rosi structure (PBD code: 3CS8) as a search model.[2] The initial model was manually built and refined with the PHENIX program package (https://www.phenix-online.org).[3] All figures were prepared using PyMOL (DeLano Scientific, San Carlos, CA, http://www.pymol.org).

Section 2. References

1 Z. Otwinowski and W. Minor, *Method Enzymol.*, 1997, **276**, 307–326.

2 R. T. Gampe Jr., V. G. Montana, M. H. Lambert, A. B. Miller, R. K. Bledsoe, M. V. Milburn, S. A. Kliewer, T. M. Willson and H. E. Xu, *Mol. Cell*, 2000, **5**, 545–555.

3 P. D. Adams, P. V. Afonine, G. Bunkóczi, V. B. Chen, I. W. Davis, N. Echols, J. J. Headd, L. W. Hung, G. J. Kapral, R. W. Grosse-Kunstleve, A. J. McCoy, N. W. Moriarty, R. Oeffner, R. J. Read, D. C. Richardson, J. S. Richardson, T. C. Terwilliger and P. H. Zwart, *Acta. Crystallogr. D. Biol. Crystallogr.*, 2010, **66**, 213–221.

Section 3. Copies of ^{1}H and ^{13}C NMR spectra of products 1-4 and VSP-77

Product 1:

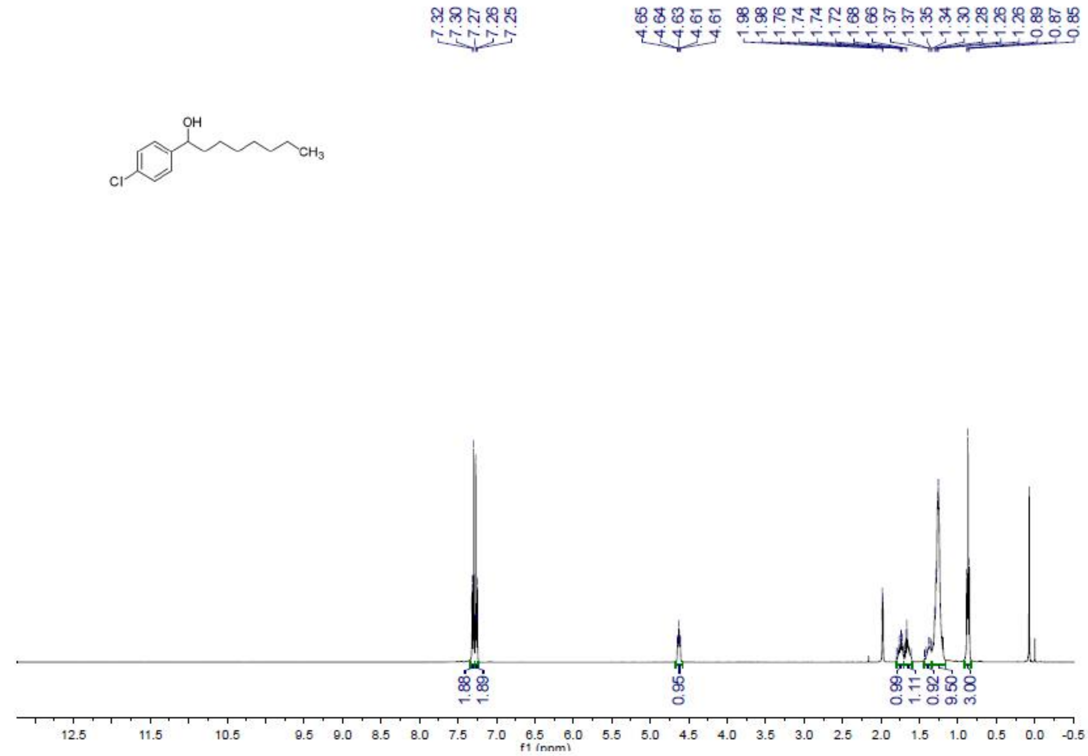

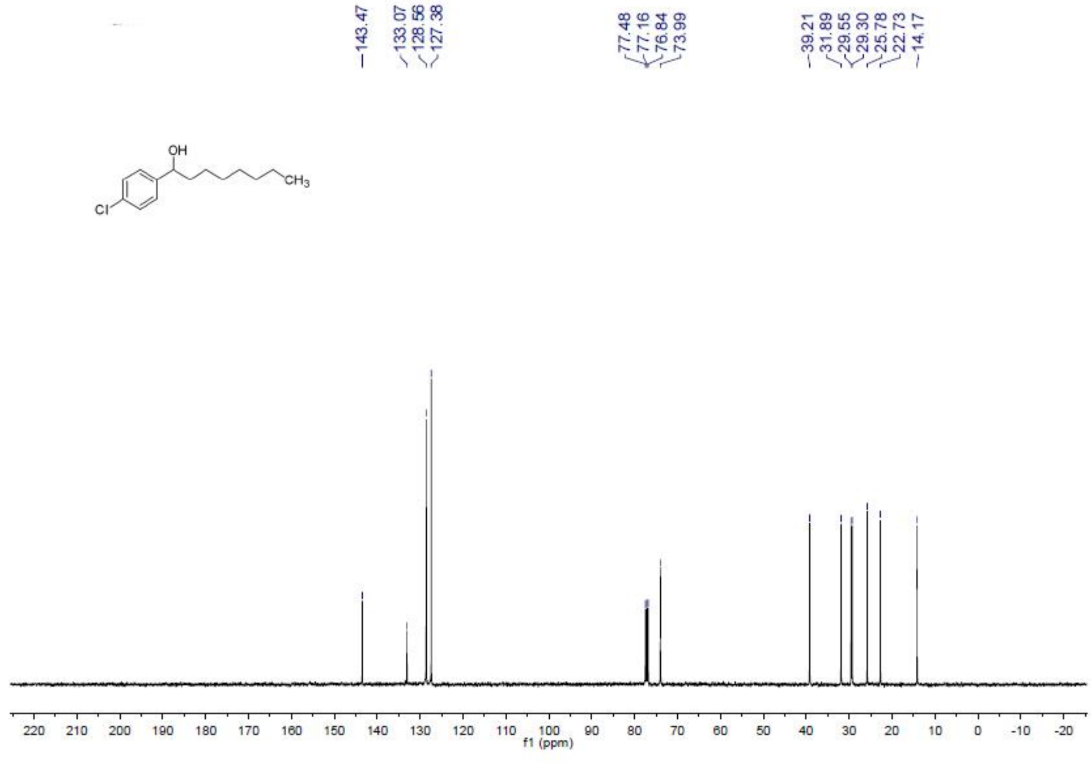

Product 2:

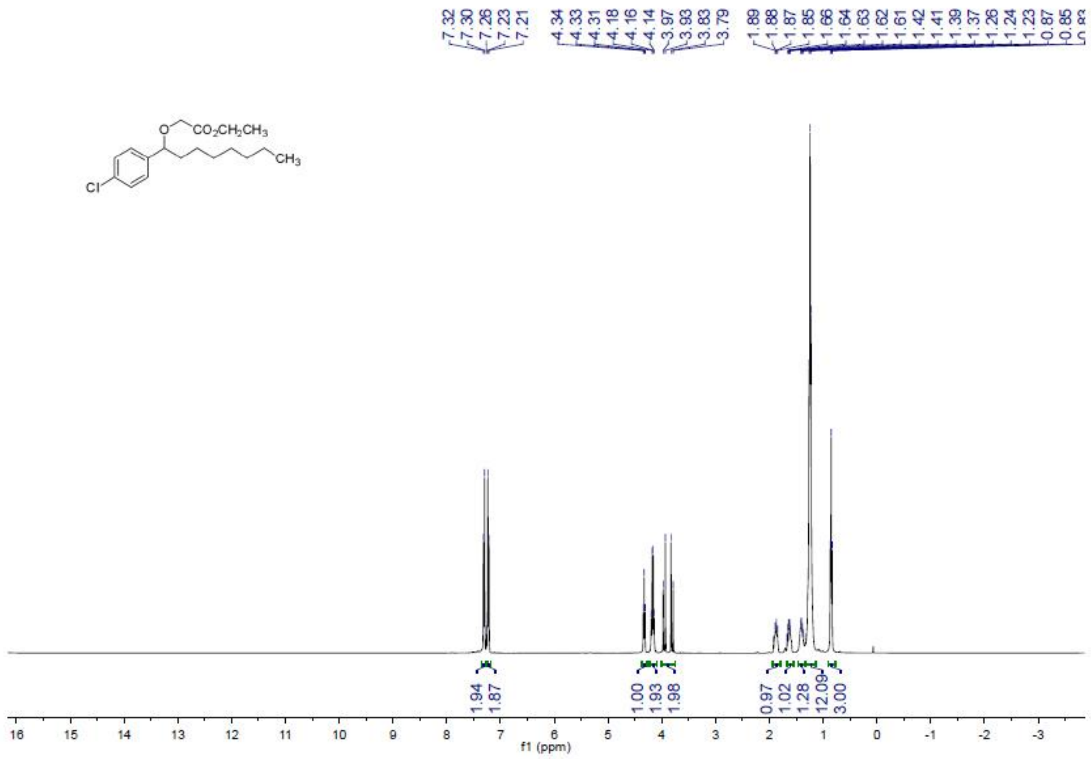

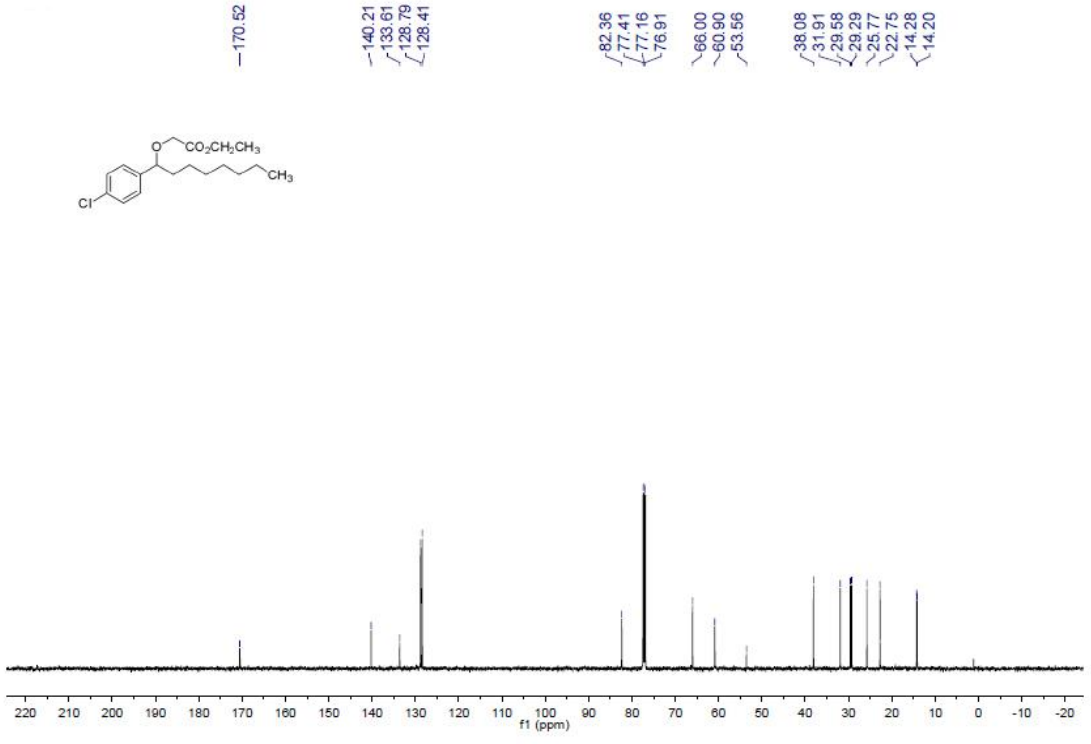

Product 3:

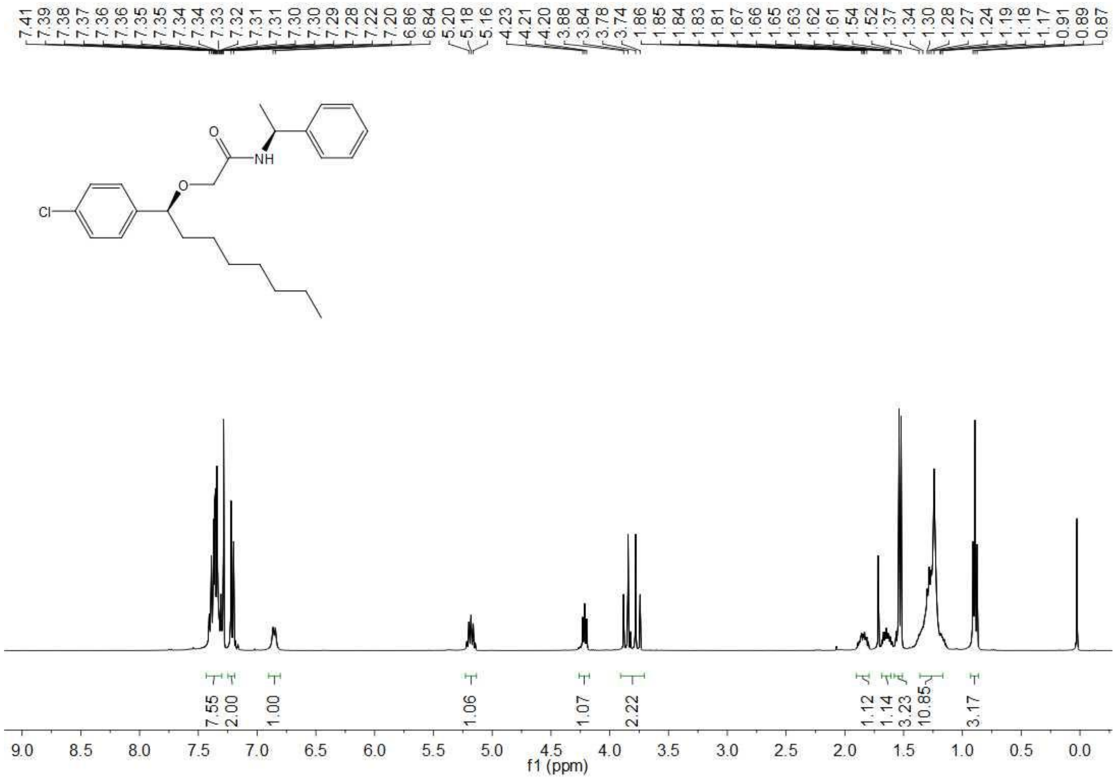

Product 4:

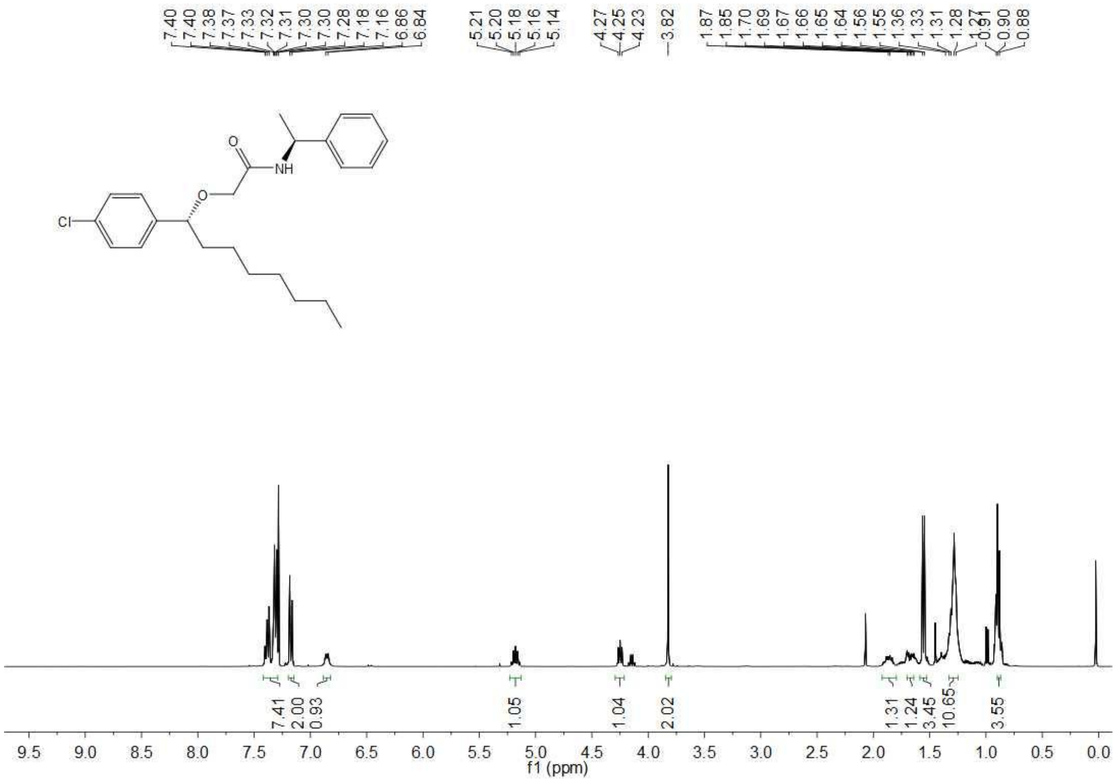

Product VSP-77:

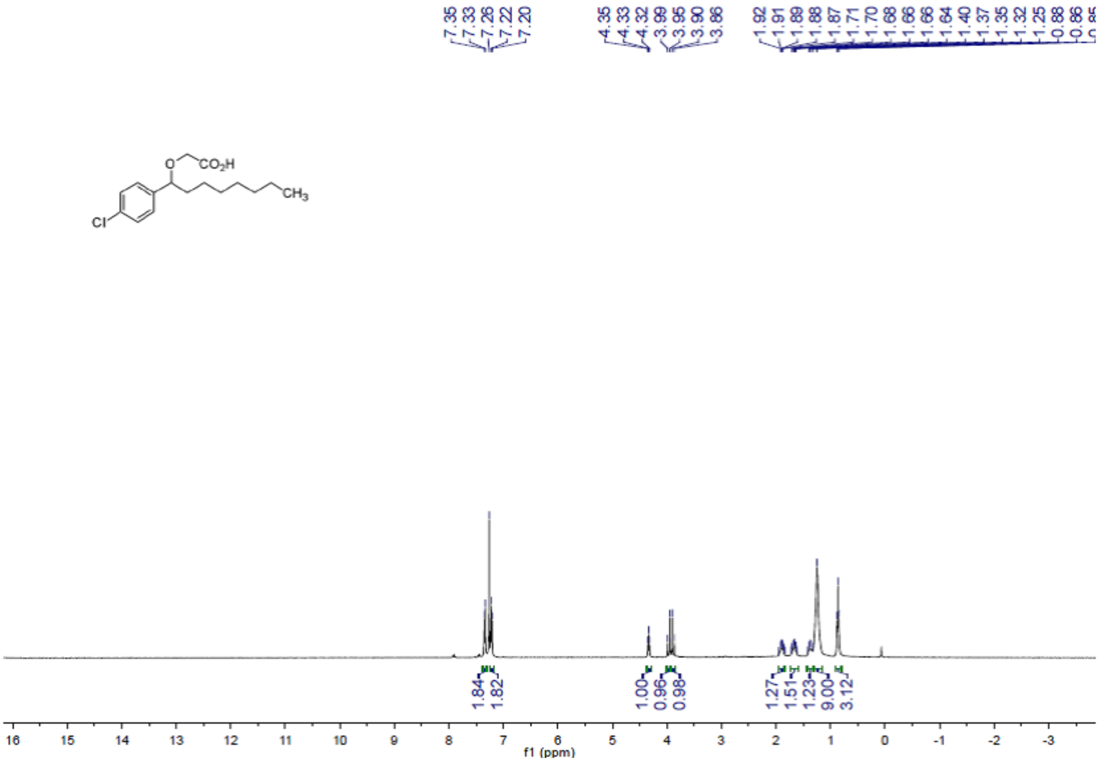

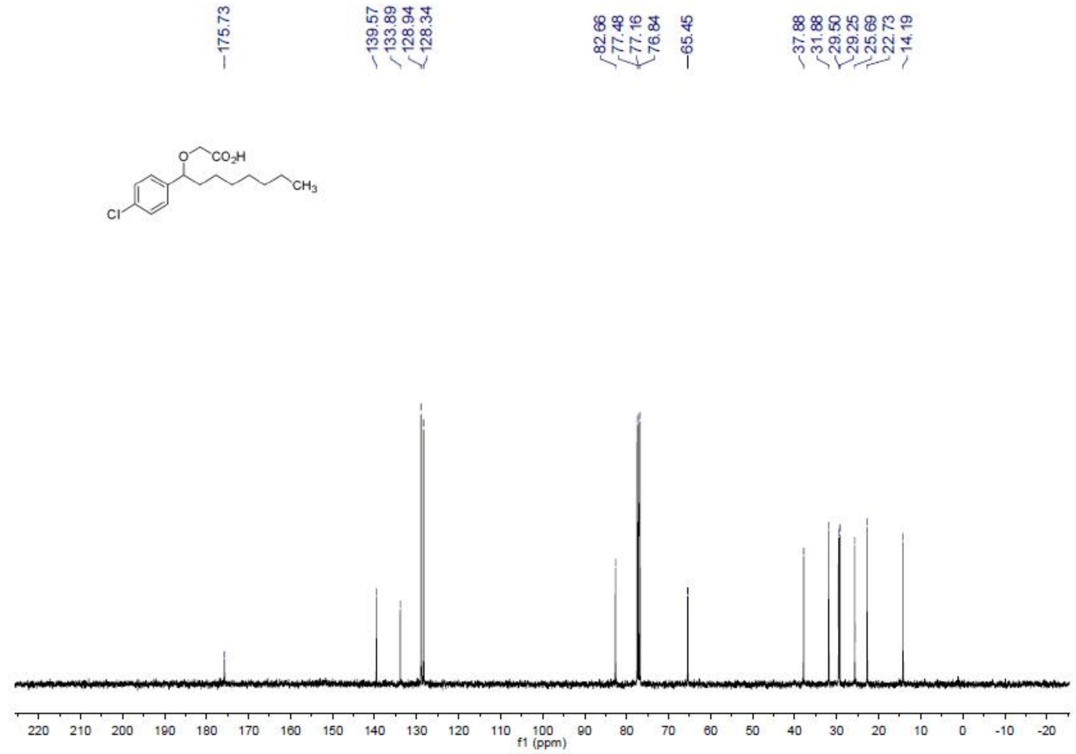

Section 4. Design of DA-based PPARγ agonists (Figure S1)

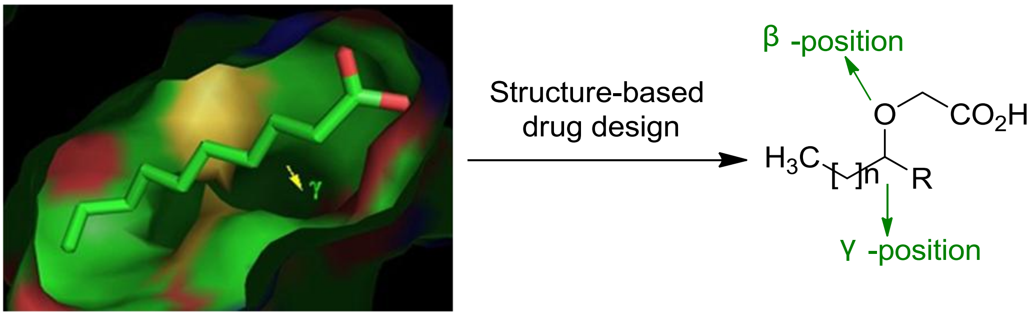

Section 5. Primer sequences used for qPCR (Table S1)

Gene	Forward primer	Reverse primer
PPARγ	GCATGGTGCCTTCGCTGA	TGGCATCTCTGTGTCAACCATG
AP2	AAGGTGAAGAGCATCATAACCCT	TCACGCCTTTCATAACACATTCC
LPL	GGGAGTTTGGCTCCAGAGTTT	TGTGTCTTCAGGGGTCCTTAG
CD36	AAGCTATTGCGACATGATT	GATCCGAACACAGCGTAGAT
FASN	GCTGGCATTCGTGATGGAGTCGT	AGGCCACCAGTGATGATGTAACTCT
C/EBPα	AGACATCAGCGCCTACATCG	TGTAGGTGCATGGTGGTCTG
Adiponectin	TGTTCCTCTTAATCCTGCCCA	CCAACCTGCACAAGTTCCCTT
Glut4	GTGACTGGAACACTGGTCCTA	CCAGCCACGTTGCATTGTAG

Section 6. Pharmacokinetic parameters for VSP-77 (Table S2)

	T_{max} (h)	C_{max} (ng/mL)	AUC_{0-t} (ng h/mL)	$AUC_{0-\infty}$ (ng h/mL)	MRT (h)	$t_{1/2}$ (h)	F (%)
ig[a]	0.500	2923	5772	5877	1.94	1.25	57.7
iv[b]	/	/	5002	5033	1.11	1.07	/

[a]Intragastric administration (oral gavage). [b]Intravenous injection.

Section 7. Inhibition Assay of VSP-77 on the hERG Potassium Ion Channel (Table S3)

compd	hERG inhibition IC_{50} (μM)
VSP-77	> 50 μM
Cisapride[a]	0.09 μM

[a]The reference drug.

Section 8. Crystal data collection and structure refinement statistics (Table S4)

	PPARγ LBD/VSP-77
Data collection	
Space group	$P2_1$
Resolution, Å	50-1.43 (1.46-1.43)
Cell parameters a, b, c, Å	43.6,53.9,66.7
α, β, γ, °	90, 107.2, 90
Total reflections	197762
Unique reflections	56829
Rsym	0.06(0.59)
I/σ	31.1 (2.3)
Completeness ,%	98.7 (97.1)
Redundancy	3.5 (3.5)
Structure refinement	
Resolution, Å	32.5-1.43 (1.46-1.43)
No. of reflections	53782
No. of residues	288
No. of solvent molecules	253
No. of non-H atoms	2492
Rwork	19.0 (32.5)
Rfree	21.6 (35.8)
RMSD bonds, Å	0.008
RMSD angles, °	1.263
Average B factor, $Å^2$	25.0
Ramachandran	
Outliers, %	0.00
Favored, %	99.26
Clash score	4.43
Rotamer outliers, %	1.62
Molprobity score	1.40
